# Modern trends in lipomodeling

**DOI:** 10.3205/iprs000108

**Published:** 2017-04-03

**Authors:** Ahmed Hassan El-Sabbagh

**Affiliations:** 1 Plastic Surgery Center, Faculty of Medicine, Mansoura University, Mansoura, Egypt

## Abstract

Lipomodeling is the process of relocating autologous fat to change the shape, volume, consistency, and profile of tissues, with the aim of reconstructing, rejuvenating, and regenerating body features. There have been several important advancements in lipomodeling procedures during the last thirty years. Four clinical steps are important for the success of engraftment: fat harvesting, fat processing, fat reinjection, and preconditioning of the recipient site. With the discovery of adipose derived stem cells and dedifferentiated cells, fat cells become a major tool of regenerative medicine. This article reviews recent trends in lipomodeling trying to understand most of the issues in this field.

## Background

Over the past 30 years, the literature has seen numerous clinical reports highlighting the benefits of autologous fat transfer [1], [2], [3], [4]. 

A greater understanding of how to maintain viable fat has led to continuous modifications in the techniques that are believed to improve the clinical outcome with reasonable long lasting results. These modifications are intended to preserve the delicate structure of adipocytes and to provide a robust blood supply on which fat cells are extremely dependent [5]. Unfortunately, there are many conflicting studies regarding the durability and integrity of autologous fat grafts [6]. 

## Methods

Electronic searches were performed using the following databases: PubMed, Ovid, Science Direct, and Oxford Journals. The following key words were searched in each of the databases: “lipomodeling”, “lipomodelling”, “fat injection”, “fat grafting”, “autologous fat grafting”, “autologous fat transfer”, “lipotransfer”, and “lipofilling”. The titles and abstracts from the initial literature search were assessed to identify articles for full-text. Also, the references section from each article was scanned manually for relevant publications.

## Results

### Definition

Lipomodeling is the process of relocating autologous fat to change the shape, volume, consistency, and profile of tissues, with the aim of reconstructing, rejuvenating, and regenerating body features.

Terms in current use to describe the technique are: micro fat grafting, fat transfer, fat injection, lipostructure, and lipofilling. 

### Lipomodeling or lipomodelling?

The origin of this word comes from the French term “lipomodelage”. It means “to give a form, to emboss”. The roots of this French word come from the Italian word “modello”, with a double “l” [7]. By searching different articles, it becomes obvious that it is written with one “l” in American English and double “l” in British English [7], [8].

### Terms

**Macrofat grafting:** fat is injected with relatively large blunt cannulas (±2 mm diameter).

**Microfat grafting:** performed in the facial area, blunt injection cannulas range from 0.7 to 0.9 mm.

**Nanofat grafting:** is combined with other modalities of microfat grafting such as sharp needle intradermal fat grafting to obtain a soft-tissue filling effect.

**Mega (large) volume fat grafting:** over 100 cc of processed fat is injected for core volume projection.

### History

In 1893, the German surgeon Gustav Neuber (1850–1932) had harvested adipose tissue from the arm and transferred it to the orbital region to correct an adherent scar resulting from osteomyelitis [9]. Neuber emphasized the importance of small grafts for more predictable results, a concept believed integral nowadays. In 1895, another German, Viktor Czerny (1842–1916), transferred a lipoma to the breast to reestablish symmetry, following unilateral partial mastectomy for fibrocystic mastitis [10].

Later, fat grafting was criticized by Peer (1950), a pioneer in the science of autologous tissue transfer, who histologically determined that fat grafts lost about 45% of their weight and mass one year after transplantation [11]. 

Interest in autologous fat transfer was rekindled again in the late 1980s. This was correlated with the widespread application of suction lipectomy by Illouz, who pioneered liposuction for body contouring [12]. In 1997, Coleman technique was introduced with good results paving the way for autologous fat to be considered as a reliable natural filler [3]. 

### Incidence

Although specific statistical reports are deficient, fat grafting is one of the most commonly performed procedures in aesthetic and reconstructive surgery. Actually, the number of fat grafting procedures for aesthetic indications in the United States has increased over 40% between 2007 and 2013 [13].

### Biology of body fat

Adipose tissue is the largest endocrine organ in the body [14]. It is composed of matrices of adipocytes interspersed with collagen fibers, stromal cells, adipose-derived stem cells (ADSC), and neurovascular structures [15], [16]. 

Throughout life, fat cells multiply in number and increase in size [17]. Individual adipocytes triple in size during the first year of life, continue to grow and multiply for the next 5 years, and grow again during adolescence, without substantial weight gain by the person [18]. After adolescence, no new adipocytes are formed. Distribution of adipose tissue changes with age. Fat in the trunk generally increases with age. The extremities tend to lose fat from the subcutaneous tissue with age, relocating to intra- and intermuscular fat deposition [19].

Brown adipose tissue (BAT) comprises less than 1% of all fat in the adult human population [20]. BAT differs significantly from white adipose tissue (WAT). BAT contains a high concentration of mitochondria and thus is exceedingly metabolically active. It has been hypothesized that the high metabolic activity of BAT may confer a protective role in metabolism and aging. The conversion of WAT to BAT and its potential metabolic benefits in adults is the focus of several studies [21].

### Fat harvesting, preparation, and reinjection

Four clinical steps appear to have a significant effect on the survival of grafted fat cells: fat harvesting, fat processing, fat reinjection, and preconditioning of the recipient site.

#### Fat harvesting

There is no difference in the viability of fat harvested from the abdomen, thigh, flank, or knee in a preclinical model [22]. These results encourage clinicians to harvest fat from the most abundant donor source thus limiting possible donor site morbidity in instances where there is a paucity of fat in a given anatomical location [23].

Usage of lidocaine with epinephrine has been reported to inhibit the growth of adipocytes in culture and slow down glucose transport. However, these findings only persisted when lidocaine was present. Once lidocaine was removed, its inhibitory effect diminished. Interestingly, more recent studies showed no difference in fat treated with infiltrative anesthetic [24]. Extrapolating these finding, one could say that the decision to use epinephrine/local anesthesia should be based on other clinical factors such as pain relief and bleeding control rather than fat cell viability.

The literature concerned with the isolated effects of negative pressure suggested that adipocytes can be suctioned below 700 mm Hg without undue trauma. Any claims of syringe suctioning being safer than machine suctioning should be carefully examined [24]. While a standard liposuction machine can generate up to one atmosphere (760 mm Hg) of negative pressure, a 60 cc syringe connected to a manometer can also generate nearly one atmosphere of negative pressure. It seems that absolute pressure and not the source of this pressure is the key variable in adipocyte trauma. The use of low pressure suction by means of larger bore cannulas appears to increase adipocyte viability.

Another variable in fat harvesting is the negative impact of air exposure. Despite its widespread mention, there is a paucity of scientific data quantifying the effect of air exposure on adipocyte viability [25]. Techniques of fat processing range from drying fat on Telfa Rolls (high air exposure) to completely closed systems employing intravenous tubing, three way stopcocks and IV bags for collection.

#### Fat processing

There is no difference in fat graft survival without treatment, with centrifugation, with washes of normal saline, with washes of lactated Ringer solution, and with combinations of centrifugation and washes [26].

In addition, there is no supporting data prevailing one process above another. Furthermore, when centrifugation is used, many articles suggested that forces greater than 3,000 rpm (1,200 g) cause more cellular damage [27], [28], [29], [30], [31], [32], [33], [34].

**RPM or g:** It is much better to use g (the capital G is not the correct unit) as a unit for centrifugation steps, which refers to the acceleration applied to your samples (so 10,000 g means 10,000 times earth's gravitational force). RPM (revolutions per minute) is not as useful a unit, because the force varies with the radius of the machine (the bigger the radius, the more acceleration applies to the samples for the same RPM).

#### Fat reinjection

Optimal cannula diameter for fat reinjection continues to be a topic of debate. One study found that viability is great with a 2.5 mm (approximately 10 to 11 gauge) cannula compared with smaller cannulas as evaluated by counting live cells using a hemocytometer under 40x magnification [35]. Some researchers concluded that increasing the diameter of aspiration and injection cannulas minimizes trauma, increases viability, and ultimately improves graft survival [36]. Similarly, another study stated that the best results were achieved with the no. 14 cannula, compared with smaller ones [37].

#### Preconditioning of recipient site

Although there are several articles that focus on improving the harvest and preparation of fat, the recipient site is often neglected when attempting to optimize the outcome. There have been studies supporting microneedling [38]. They mentioned that there was more vascularity, higher graft survival, and better graft integrity with less fibrosis after preconditioning with microneedling 1 week before grafting [39].

### Adipose stem cells and fat graft enhancement

Now we are in the era of regenerative medicine. A new type of stromal cells similar to fibroblast were identified showing multipotent differentiation potential, and these new cells proved to be a kind of adult stem cells through various scientific experiments [40], [41]. These adult stem cells extracted from adipose tissue have various names such as adipose tissue derived stem cells, adipose-derived stromal cells (ADSC), adipose tissue-derived mesenchymal stromal cells, or adipose stromal cells (ASC), but recently, it is arranged as ADSC or ASC. 

ASC can not only be obtained easily and plentifully from lipoaspirate but also be differentiated into various tissues such as adipocytes, cardiomyocytes, chondrocytes, endothelial cells, myocytes, neuron-like cells, and osteoblasts [41]. 

In ischemic condition, ASC uniformly differentiates into neoangiogenesis, which can be helpful for treating postoperative wound problems, radiation necrosis, or ischemic flap [6]. A new type of fat grafting called cell-assisted lipotransfer introduced after discovery of ASC is known as a good method for increasing the survival rate of fat and for reducing side effects in comparison with existing methods [42], [43].

Treatment of fat grafts with p38 inhibitor would (a) prevent apoptosis of ASC in the fat grafts, (b) increase ASCs' proliferation, and (c) stimulate the release of several angiogenic factors and promote revascularization [44].

Another method to increase the survival rate of fat grafting is the use of platelet-rich plasma (PRP). It is known that various cytokines released from platelets promote healing process and improve fat graft survival [45], [46].

### Cryopreservation

Adipose-derived stem cells (ADSC), either isolated from fresh adipose tissues before ADSC cryopreservation or isolated from cryopreserved fat tissues, showed unaltered capabilities of proliferation and differentiation after optimal cryopreservation protocols. This confirms the probability of ADSC in providing an important source for cell-based therapy and tissue engineering [47], [48]. Successful cryopreservation of adipose tissue and ADSC can lead to a new era in fat grafting and ADSC-related tissue regeneration therapy in plastic surgery [49], [50], [51], [52], [53].

### Fate of the graft 

Scientific understanding about engraftment processes is still insufficient. According to Eto and his colleagues, adipocyte existing within 300 µm from the surface of transplanted adipose tissue survives. However, most of the adipocytes that are located deeper in transplanted fat die within 24 hours. At this moment, some ASC survive in the deeper part of the transplant and play an important role to regenerate adipose tissue in transplanted fat [54]. 

Coleman has suggested that micrografts of “seeded fat” get vascularized within muscle or subcutaneous tissue and Guerrosantos stated that fat stem cells from fat grafting recruit other cells into the recipient site [3], [4]. Animal studies have documented fat resorption rates of 60–70%, with many incorporating objective measures of assessment. Clinically, the majority of physicians reported short-term patient satisfaction to be excellent to good and long-term patient satisfaction to be good to fair [12].

### Quantifying fat viability

Autologous fat presents ample opportunities for clinical use and innovation, and investigation into fat graft injection location indicates that there is no statistically significant difference in neovascularization signals between the subcutaneous plane and the local fat pad in the athymic rat model [55]. 

There is no standard method to determine fat viability or volume augmentation after grafting. Several modalities for assessing breast volume (BV) after fat injection have been published, including water displacement, imprint casts, 3D photography, computed tomography, and magnetic resonance imaging (MRI) [56], [57]. Three-dimensional photography has gained popularity because of its speed and accessibility [58], [59]. However, this technique is clearly inferior to MRI because of its lower accuracy [60], [61], [62], [63].

Also, fat graft viability has been estimated through visual assessment, membrane integrity staining and conventional histology, special staining for apoptosis or mitochondrial function, histologic analysis for cell death, colorimetric salt viability assay (i.e., XTT assay), cell count per high-power field, biochemical assays, three-dimensional laser scanning, and others [64].

### Guidelines for training

Lipomodeling is relatively new, yet considerable expertise has already been developed in some institutions amongst plastic surgeons. 

The aim for these guidelines is to minimize clinical risk for patients and demonstrate competence for practitioners. As with all new procedures, formal training must be undertaken and should have the following components:

- Background theory and knowledge, including indications and complications
- Practical skills
- Arrangements for supervision, assistance, and mentoring during local implementation (log book)
- A whole-team approach involving the multidisciplinary team including theatre staff
- Evidence of completion of training to an acceptable standard before starting practice (exams)

### Preparation of the patient

#### Informed consent

Informed consent is an ongoing agreement with the patient about his treatment and requires full and adequate explanation of the risks and the benefits. It should be written, preferably illustrated and the information process should be documented in the medical record [65], [66], [67]. 

#### Pre-operative assessment

- Current use of medications such as aspirin, non-steroidal anti-inflammatory drugs, cytotoxic and immunosuppressant drugs due to associated risks of bleeding and infection.
- Availability of adequate donor sites; there must be appropriate donor sites for fat transfer without causing damage to the underlying structures or deformity.
- Suitability of the recipient site [68].

#### Specific considerations in patients with previous breast cancer

Breast cancer patients should have an initial follow-up mammogram before commencing lipomodeling [68].

### Clinical applications of fat grafting

#### 1) Skull reshaping

Head molding with fat grafting is easier than with bone grafting because the desired shape can be obtained by altering the injection volume and location [69].

#### 2) Face

Fat grafting has been frequently used as an adjunct procedure in the treatment of congenital conditions, facial aging, and facial reconstruction. In these cases, there is a loss of subcutaneous facial volume that results in unfavorable changes in skin integrity and makes the underlying bony structure more prominent. Although rhytidectomy techniques address the facial skin envelope and underlying fascial layer, patients frequently require an additional volume for a more youthful look. Common sites of fat transfer for facial rejuvenation include those that atrophy with age, such as the periorbital region, malar region, marionette lines, prejowl sulcus, and lips [70].

#### 3) Secondary rhinoplasty

In patients who have undergone multiple operations, microinjection of adipose tissue can be a simple and reliable alternative for correction of imperfections following rhinoplasty [71].

#### 4) Cleft lip

A successful cleft lip repair relies on the understanding of anatomical segments and margins. Even under the best circumstances, an obvious scar, contour irregularities, and asymmetries may persist [72].

Infant fat produces more biologically robust adipose-derived stem cells than adult tissue does. Immediate fat grafting may be a promising strategy to improve lip appearance, contour, and scarring during primary cleft lip repair [73].

#### 5) Velopharyngeal incompetence

Autologous (lipofilling) pharyngeal wall augmentation has been successfully described. Contouring of palatal bulk can better be achieved through the lipoinjection technique than with a larger palatal V-Y pushback or standard pharyngeal flap. Lipoinjection of the palate can be performed as an outpatient procedure with only minor discomfort regarding the donor and operative sites [74].

#### 6) Vocal cord paralysis

Autologous fat has been effective in laryngeal surgery for treatment of unilateral vocal fold paralysis [75].

#### 7) Breast

Breast augmentation surgery using cell-assisted lipotransfer has increased greatly [76], [77]. Lipomodeling has been used for Poland syndrome, partial mastectomy or lumpectomy to improve breast shape [78], [79]. Many patients after breast reconstruction with flap operation or breast prostheses want fat grafting to improve the breast shape or to substitute breast prostheses for autologous fat. Also, there is a new method using BRAVA, an external pre-expansion device to enlarge the skin pocket before fat grafting to increase the survival rate of fat [80].

#### 8) Burns

It is reported that the application of fat graft to a burn wound or extensive wound not only reduces the therapy period but also reduces deformity at the end of the healing process [81]. In addition, lipomodeling is conducted to improve scar and scar contracture by injury or burn [82], [83].

#### 9) Hand

##### Rejuvenation

Lipomodeling has been used for rejuvenation of the dorsum of the hand and the fingers. By minimizing the number of injection sites, bruising is reduced considerably without compromising accessibility to the entire hand [84], [85].

Lipomodeling of the fingers is associated with an increase in the diameter of the PIP joint. Careful consideration of the third and fourth fingers is necessary, because many patients wear rings on these fingers [86].

##### Dupuytren contracture

Hovius had performed percutaneous aponeurotomy for Dupuytren disease with simultaneous fat grafting to prevent recurrent contracture through introduction of new soft tissue. Success rates were comparable to those of standard needle aponeurotomy but with improved soft-tissue quality. Unfortunately, long-term rates of recurrent Dupuytren contracture were not measured [87].

##### Tenolysis

Lipografted tenolysis may be a promising alternative method for treating post-traumatic and post-surgical tendon adherence [88].

#### 10) Peristomal hernia

The use of lipomodeling to correct peristomal soft tissue volume deficiency of an ileostomy has been previously reported and shows some promising results [89], [90]. Lipomodeling improves the peristomal contour, allowing better adhesion of the stoma bag to the abdominal wall. A better fit of stoma reduced leakage, resulting in an improved quality of life to the patient [91].

#### 11) Genetalia

##### Phalloplasty

Augmentation phalloplasty of the normal penis is gaining popularity among a subgroup of men and is performed not only by plastic surgeons but also by urologists, “cosmetic surgeons”, and general surgeons [92].

Fat injection is commonly performed for penile augmentation. The injection plane is usually between Bucks’ and Dartos’ fascia. Complications following this procedure can result from inadequate blood supply, fat necrosis, fat resorption, and fat migration [93].

##### Stress incontinence

Periurethral injection of autologous fat has been documented in several studies [94], [95]. The reported success rate ranged from 23 to 57% [96]. It is used for primary genuine stress incontinence, especially for those patients who have low urethral resistance. Also, it is reserved for patients in whom surgery has failed and who are unsuitable for major operations [97].

##### Pain after episiotomy

Lipomodeling also seems to be a promising treatment for correction of perineal/vaginal scars after episiotomy and perineal laceration. It is well tolerated and offers encouraging results, with improvement of pain and better sexual function [98], [99].

#### 12) Scleroderma

Scleroderma is a chronic systemic autoimmune disease characterized by microvascular abnormalities and progressive skin and internal organ fibrosis [100]. By combining the lipomodeling with the regenerative cell therapy tissue, graft longevity and positive effect could be enhanced. In scleroderma, applications could concern both hands and face. For hands, the association of both ischemic manifestations and mechanic ulcers related to skin tightening on bone could be relieved. For the face, optimization of the regenerative effect of autologous fat grafting could be achieved [101].

#### 13) HIV

Fat depots redistribution in the HIV-associated lipodystrophy includes visceral fat accumulation in the abdomen, subcutaneous fat accumulation in breasts and in the cervical and dorsal area (buffalo hump) with fat wasting in the legs, arms, buttocks, and face [102], [103]. Lipomodeling is essential to ameliorate lipoatrophy facial stigmata, giving back fullness to the faces. Autologous tissue is preferred for facial enhancing when possible [104], [105].

#### 14) Polio sequelae

Fat injection procedure can be a good technique to use with patients who have polio sequelae, in particular, patients with short legs and volume deformities. Fat grafts have to be harvested and applied with thick cannula, and the volume of the fat injection must not be exagerated in each session. With these measures, the results will be longer-lasting and more effective [106].

#### 15) Foot ulcers

Chronic ulceration of the foot is one of the most challenging conditions to treat for plastic surgeons. Predisposing conditions such as diabetes or peripheral vascular disease commonly hinder the wound healing. The lipoaspirate may serve as a matrix for new cells to migrate and to promote neovascularization and granulation tissue [107].

### Complications of lipomodeling

Accidental injection of fat into the arterial system can result in catastrophic complications. Blindness and stroke have occurred with the usage of sharp needles or sharp cannulas [108], [109], [110], [111].

Another major complication after fat grafting is oil cyst formation and calcifications. Oil cysts show never-ending inflammation and progressive calcification in the cyst wall, which can induce many types of clinical complaints including unmanageable infection and pain. Oil cysts are a typical result of roughly done fat injection, and surgeons should avoid any bolus injection and introduce noodle-like columns of fat or small aliquots (pearls) of fat [112].

Small fat necrosis induces a fibrous deposit, which later develops into a sand-like micro/macro-calcification over years. This type of calcification usually shows no clinical symptoms, though the latter can be troublesome. When high numbers of sand-like and/or egg shell-like calcifications are detected in the mammogram, they may interfere with a detailed evaluation of mammographic images and a precise diagnosis of breast cancer cases [113].

Although infection is uncommon with fat grafting, infection following fat necrosis or hematoma can occur. As the grafted fat is not vascularized, it can be a focus of infection once contaminated by bacteria. Strict sterile technique should be done. The use of intraoperative antibiotics is recommended, but perioperative use of antibiotics is not recommended unless there is a specific indication [114].

In the learning curve of fat injection to the breast, it is rare but possible to induce pneumonia. Pneumonia is induced by damaging the pleura with an injection cannula/needle. Pneumonia is usually first recognized as a complaint of chest pain in the next morning and can be diagnosed by monitor of oxygen saturation, chest X-ray, and/or CT scan [115].

Aesthetic complications, such as placement of too much or too little fat in a specified area are not uncommon. Irregularities after fat grafting diminish significantly as the surgeon gains experience [116].

One of the most difficult tasks for the surgeon is preparing patients to expect the bruising and swelling. Elevation, cold therapy, and external pressure with elastic tape may speed recovery. The patient is asked to avoid heavy pressure on the grafted areas for 7 to 10 days to avoid migration of the grafted fat [117].

Many patients find the removal of fat and the body contouring performed at the same time to be advantageous, yet even a surgeon who is expert in liposuction may produce liposuction deformities. Furthermore, some patients simply do not have adequate donor sites, especially if they have previously undergone liposuction. Complications of the donor sites are rare, but irregularity of the surface could occur, particularly when an excessive volume liposuction is performed in skinny patients [118].

### Non-surgical lipomodeling

Redistribution of fat from the thigh to the breast with a topical lipolytic cream has been reported. Women with lower body fat distribution often have smaller breasts and desire smaller thighs and larger breasts. Reduction of body fat through dieting mobilizes fat preferentially from the upper body in these women. Using a topical lipolytic cream on the thighs during weight loss may counteract upper body preferential fat loss that is often distressing to women with lower body fat distribution [119]. Topical aminophylline cream may represent a less invasive, reversible, and potentially less costly method of thigh fat reduction that is associated with fat redistribution to the breasts [120].

### Future

Future fat grafting may be more like fat tissue engineering incorporating adipocytes, ASC and adequate scaffold materials. Cell-assisted lipotransfer could be upgraded by enrichment with adequate number of cultured ASC which significantly affect the engraftment [121].

Full-scale clinical application of stem cell therapy is delayed by legal control to ensure patients’ safety, but the effect of stem cell therapy has been already proved through scientific verification during the past decade [122]. 

The business of stem cell bank has already started by ambitious investors, but it is too early to create a profit yet. It may not be easy for ordinary people to spend a large amount of money to preserve their own stem cells in advance for unrealized further medical techniques [123]. 

## Conclusion

Scientific knowledge about fat and fat tissue transfer is updated continuously. Fat presents a unique regenerative complex as well as a high energy resource related to tissue repair and regeneration. Although many techniques have been described, there have been no meticulously controlled comparative trials to truly determine which method may be the standard. 

## Notes

### Competing interests

The author declares that he has no competing interests.

### Ethical standards

This study has been performed in accordance with the ethical standards set forth in the 1964 Declaration of Helsinki and its later amendments.
